# Elevated levels of serum CDCP1 in individuals recovering from severe COVID-19 disease

**DOI:** 10.18632/aging.203898

**Published:** 2022-02-16

**Authors:** Jose-Ramon Blanco, María-Jesús Cobos-Ceballos, Francisco Navarro, Isabel Sanjoaquin, Carlos Armiñanzas, Enrique Bernal, Luis Buzon-Martin, Miguel Viribay, Laura Pérez-Martínez, Simona Espejo-Pérez, Borja Valencia, Jesus Guzman-Aguilar, Juan-Jose Ruiz-Cubillan, Consuelo Alcalde, Fernando Gustavo Gutierrez-Herrero, Julian Olalla, Eva-Maria Andres-Esteban, Bernabe Jurado-Gamez, Javier Ugedo

**Affiliations:** 1 Servicio de Enfermedades Infecciosas, Hospital Universitario San Pedro, Logroño, La Rioja, Spain; 2 Centro de Investigación Biomédica de La Rioja, Logroño, La Rioja, Spain; 3 Instituto Maimónides de Investigación Biomédica de Córdoba, Universidad de Córdoba, Córdoba, Spain; 4 Servicio de Neumología, Hospital Universitario Reina Sofía, Córdoba, Spain; 5 Servicio de Medicina Interna, Hospital Costal de Sol, Marbella, Málaga, Spain; 6 Servicio de Enfermedades Infecciosas, HCU Lozano Blesa, Zaragoza, Spain; 7 Servicio de Enfermedades Infecciosas, H Universitario Marqués de Valdecilla, Santander, Spain; 8 Unidad de Enfermedades Infecciosas, Hospital General Universitario Reina Sofía de Murcia, Murcia, Spain; 9 Servicio de Medicina Interna, Hospital Universitario de Burgos, Burgos, Spain; 10 Vitro Laboratory, Madrid, Spain; 11 Servicio de Radiología, Hospital Universitario Reina Sofía, Córdoba, Spain; 12 Servicio de Neumología, Hospital Costal de Sol, Marbella, Málaga, Spain; 13 Servicio de Neumología, HCU Lozano Blesa, Zaragoza, Spain; 14 Servicio de Neumología, H Universitario Marqués de Valdecilla, Santander, Spain; 15 Servicio de Neumología, Hospital General Universitario Reina Sofía de Murcia, Murcia, Spain; 16 Servicio de Neumología, Hospital Universitario de Burgos, Burgos, Spain; 17 Grupo PBM, Instituto de Investigación-IdiPaz, Madrid, Madrid, Spain; 18 Universidad Rey Juan Carlos, Madrid, Madrid, Spain; 19 Servicio de Neumología, Hospital Universitario San Pedro, Logroño, La Rioja, Spain

**Keywords:** biomarkers, CDCP1, recovery, COVID-19

## Abstract

Background: COVID-19 survivors report residual lung abnormalities after discharge from the hospital. The aim of this study was to identify biomarkers in serum and induced sputum samples from patients after hospitalization for COVID-19.

Methods: Patients admitted to hospitals in Spain with laboratory-confirmed COVID-19 were recruited for this study. SARS-CoV-2-infected patients were divided into groups with mild/moderate and severe disease according to the severity of their symptoms during hospitalization. Levels of 92 biomarkers were measured in serum and induced sputum samples.

Results: A total of 108 patients (46.2% severe cases) were included in this study. The median number of days after the onset of symptoms was 104. A significant difference was observed in diffusing capacity for carbon monoxide (DLCO), an indicator of lung function, whereby DLCO <80% was significantly lower in severe cases (p <0.001). Differences in inflammatory biomarkers were observed between patients with mild/moderate and severe disease. For some biomarkers, correlations in serum and induced sputum levels were detected. Independent predictors of severe disease were DLCO <80% and the serum CDCP1 value.

Conclusions: Higher levels of CDCP1 remain after hospital discharge and are associated with the severity of COVID-19. The possible prognostic implications warrant further investigation.

## INTRODUCTION

According to the Johns Hopkins Coronavirus Resource Center, more than 156 million people worldwide have been infected with SARS-CoV-2 during the COVID-19 pandemic [1]. Although COVID-19 may cause multiple organ damage, pneumonia is the most frequent manifestation, ranging in severity from asymptomatic cases to cases of critical respiratory failure [2]. Furthermore, prospective studies have shown that the effects of COVID-19 continue after resolution of the symptoms of acute infection [3]. Thus, prospective studies related to outcomes following recovery from COVID-19 might improve our understanding of this disease, its sequelae, and possible interventions to improve this situation. Indeed, it is not well known whether the severity of the disease is associated with a persistent abnormal inflammatory state.

In other diseases caused by coronaviruses, such as severe acute respiratory syndrome (SARS) and Middle East respiratory syndrome (MERS), higher levels of proinflammatory cytokine were observed during the acute phase, and severe lung lesions developed [4, 5]. In the current COVID-19 pandemic, residual lung abnormalities have been observed at 1-3 months after discharge from the hospital though information about serum inflammatory status during recovery from COVID-19 is limited. The identification of indicators of a post-COVID inflammatory state might improve the clinical management of these patients. Our objective was to assess a broad panel of markers in serum and induced sputum samples from individuals who had recovered from COVID-19; we compared the markers not only between groups but also between samples types.

## MATERIALS AND METHODS

A description has been published elsewhere [6]. Briefly, this was a prospective study of patients older than 18 years who were admitted to different hospitals in Spain with COVID-19 confirmed by a real-time PCR (RT-PCR) assay for SARS-CoV-2.

Patients were divided into groups based on whether they had had mild (including mild and moderate) or severe disease [7]. Patients who needed invasive mechanical ventilation were excluded because of its impact on systemic inflammation [8]. The epidemiological history, medical history, comorbidities, chronic treatments, and laboratory parameters of the patients were evaluated. Pulmonary function testing, the standardized 6-minute walk test (6MWT) [9] and chest-computed tomography (CT) were performed at least 45 days after symptom onset. CT findings were considered normal if ground-glass opacities, the crazy-paving pattern, consolidation or linear opacities were absent [10].

The exclusion criteria included prior need for invasive mechanical ventilation, chronic infectious diseases, chronic lung diseases, concurrent autoimmune or malignant diseases, chronic use of corticosteroids or immunosuppressive therapies, pregnancy, alcohol/drug abuse, or a condition that did not allow participation in this study. The study was approved by the Institutional Research Ethics Committees. All participants provided written informed consent.

Serum samples were obtained from blood drawn during a study visit and stored at -80° C. Sputum was induced as previously described [11] and stored at -80° C. Ninety-two inflammation-related proteins were analyzed in the serum and sputum samples using the Olink Inflammation panel (Olink Proteomics, Uppsala, Sweden; Supplementary Table 1). In short, the method was based on proximity extension assay technology: 92 antibody probe pairs were bound to their specific target protein, forming a polymerase chain reaction target sequence through proximity-dependent DNA polymerization that was detected and quantified using a standard real-time polymerase chain reaction [12]. The output was normalized in 2 steps and presented as the relative semiquantitative normalized protein expression (NPX) unit. Finally, the normalized protein expression data were log_2_ transformed. For sputum samples, the hook effect was ruled out after analyzing undiluted and diluted samples. Protein interactions and biological functions were investigated using the STRING database [13].

Serum angiotensin-converting enzyme 2 (ACE2) levels were measured by a human enzyme-linked immunosorbent assay kit (ELISA) (Invitrogen). The procedure was performed according to the manufacturer’s instructions.

### Data analysis

Categorical variables are reported as frequencies and proportions. Continuous variables are presented as the medians (interquartile ranges [IQRs]; p25, p75). To compare demographic and clinical variables between groups, the chi-square test or Fisher's exact test was used for each categorical variable, as appropriate. For quantitative variables, the nonparametric Mann-Whitney U test was employed. Linear regression was also performed. Multivariate logistic regression analyses (with backward stepwise elimination) were carried out. We entered into the model variables associated in bivariate analysis with a p-value <0.20, excluding those that were collinear with other factors. Statistical significance was set at p <0.05, and the statistical analyses were performed using SPSS 24.0 software (SPSS Inc., Chicago, IL, USA). Graphics were generated with GraphPad Prism 6 software (GraphPad Software, La Jolla, CA, USA).

## RESULTS

A total of 108 patients were included in this study. Of them, 46.2% had a severe COVID-19. Table 1 shows the patient characteristics according to COVID-19 severity. Statistical differences were observed when comparing male sex and body mass index. However, no differences with regard to smoking history, days of hospitalization or other comorbidities or chronic treatments were found.

**Table 1:** Characteristics of COVID-19 survivors according to disease severity.

	**All of them (n = 108)**	**Mild/moderate (n = 58)**	**Severe (n = 50)**	***P* value**
Age in years, median (p25; p75)	55.0 (49.0; 62.0)	53.5 (45.0; 61.0)	56.5 (51.0; 63.0)	0.092
**Male sex, n (%)**	**69 (63.9)**	**31 (53.4)**	**38 (76.0)**	**0.015**
Caucasian, n (%)	94 (87.0)	50 (86.2)	44 (88.0)	0.782
Never smoker history, n (%)	66 (61.1)	40 (69.0)	26 (52.0)	0.071
**BMI, median (p25; p75)**	**27.9 (25.9; 30.9)**	**27.4 (24.5; 30.1)**	**28.0 (26.1; 31.2)**	**0.033**
**Comorbidities**				
Cardiovascular disease, n (%)	5 (4.6)	2 (3.4)	3 (6.0)	0.661
Hypertension, n (%)	27 (25.0)	11 (19.0)	16 (32.0)	0.119
Diabetes mellitus, n (%)	12 (11.2)	3 (5.3)	9 (18.0)	0.062
Chronic renal failure, n (%)	2 (1.8)	0 (0)	2 (4.0)	0.212
Chronic aspirin use, n (%)	4 (3.7)	1 (1.7)	3 (6.0)	0.241
Chronic statin use, n (%)	12 (12.0)	5 (8.6)	8 (16.0)	0.240
Chronic ACE/ARA-II use, n (%)	19 (17.5)	8 (13.8)	11 (22.0)	0.264
**SARS-CoV-2 data during hospitalization admission**				
**Days of hospitalization,** median (p25; p75)	7.5 (6.0; 10.0)	8.0 (6.0; 10.2)	7.0 (5.6; 10.0)	0.975

Note: ACE, angiotensin converting enzyme inhibitors; ARA-II, angiotensin II receptor blockers; BMI, Body mass index; DLCO, diffusion capacity of the lung for carbon monoxide; SD, Standard deviation.

Table 2 shows the analytical and biomarker parameters according to COVID-19 severity. Significant differences in serum levels of glucose and creatinine were detected but no differences in ACE2. In relation to biomarkers, only 6 serum and 1 sputum biomarkers showed differences. All of them, except C-X3-C motif chemokine ligand 1 (CX3CL1), were elevated in patients with severe disease. After analyzing serum biomarkers, direct interactions between interleukin (IL)-18 and CX3C chemokine receptor 1 (CX3CL1), especially IL6 with both IL18 and osteoprotegerin (OPG, also known as TNFRSF11B), were predicted by STRING analysis (Supplementary Figure 1).

**Table 2:** Analytical and biomarker characteristics among COVID-19 survivors according to disease severity.

	**All of them (n = 108)**	**Mild/moderate (n = 58)**	**Severe (n = 50)**	***P* value**
**Serum parameters**, median (p25; p75)				
WBC count, cells/μL	6.1 (5.1; 6.6)	6.01 (5.0; 6.5)	6.2 (5.3; 6.7)	0.299
**Glucose, mg/dL**	**95.0 (90.0; 111.0)**	**93.0 (87.5; 101.0)**	**100.5 (94.0; 117.7)**	**0.002**
**Creatinine, mg/dL**	**0.8 (0.7; 0.9)**	**0.8 (07; 0.9)**	**0.9 (0.8; 1.0)**	**0.048**
ACE2, ng/mL	2.4 (0.8; 11.4)	2.5 (0.7; 12.2)	2.2 (0.8; 10.8)	0.803
ALT, UI/L	22.0 (17.0; 33.0)	24.0 (16.0; 33.0)	22.0 (17.0; 32.2)	0.915
AST, UI/L	22.0 (19.0; 26.0)	23.0 (19.0; 27.0)	22.0 (19.7; 32.2)	0.268
LDH, UI/L	189.0 (170.0; 218.0)	187.0 (171.0; 225.5)	193.5 (168.5; 216.5)	0.891
CRP g/dL	3.3 (1.0; 4.0)	3.9 (1.0; 4.0)	3.1 (1.0; 4.0)	0.875
**Serum biomarkers**, median (P25; p75)				
**CDCP1**	**2.5 (2.0; 2.9)**	**2.3 (2.0; 2.8)**	**2.8 (2.3; 3.0)**	**0.001**
**OPG**	**9.9 (9.7; 10.0)**	**9.8 (9.7; 10.0)**	**10.0 (0.8; 10.1)**	**0.034**
**IL6**	**2.0 (1.7; 2.5)**	**1.9 (1.6; 2.4)**	**2.3 (1.8; 2.7)**	**0.014**
**IL15RA**	**1.3 (1.1; 1.4)**	**1.2 (1.1; 1.4)**	**1.3 (1.1; 1.5)**	**0.011**
**IL18**	**8.6 (8.3; 8.9)**	**8.5 (8.2; 8.8)**	**8.6 (8.5; 9.2)**	**0.005**
**CX3CL1**	**4.0 (3.7; 4.2)**	**3.9 (3.7; 4.1)**	**3.1 (3.8; 4.4)**	**0.036**
**Sputum biomarkers**, median (P25; p75)				
**MCP3**	**0.7 (0.4; 1.4)**	**0.7 (0.4; 1.2)**	**0.9 (0.5; 2.3)**	**0.039**

Biomarkers are presented as median of the NPX values. Note: ACE2, angiotensin-converting enzyme 2; ALT, Alanine aminotransferase; AST, Aspartate aminotransferase; CDCP1, CUB domain-containing protein 1; CRP, C-reactive protein; CX3CL1, Fractalkine; IL6, Interleukin 6; IL15RA, Interleukin 15 receptor subunit alpha; IL18, Interleukin 18; LDH, Lactate dehydrogenase; MCP3, Monocyte chemotactic protein 3; OPG, Osteoprotegerin; WBC, White blood cell count.

Table 3 provides information about the tests that were performed. A lower DLCO value, a lower 6MWT distance and pathologic CT findings were significantly associated with the severe COVID-19.

**Table 3:** Pulmonary function test and computed tomography among COVID-19 survivors according to disease severity.

	**All of them (n = 108)**	**Mild/Moderate (n = 58)**	**Severe (n = 50)**	***P* value**
**Days elapsed from symptom onset to test performance**, median (p25; p75)	104.0 (90.5; 125.0)	104.5 (94.7; 127.0)	103.0 (88.5; 121.2)	0.273
**Functional lung parameter and imaging CT**				
FVC (%), median (p25; p75) FVC >80%, n(%)	106.1 (93.2; 114.0)	105.0 (95.0; 114.5) 53 (91.4)	107.6 (91.0; 113.0) 47 (94.0)	0.671 0.722
FEV1 (%), median (p25; p75) FEV1 >80%, n (%)	104.5 (95.0; 113.5)	103.0 (94.7; 119.0) 54 (93.1)	105.0 (95.0; 113.0) 48 (96.0)	0.651 0.684
FEV1/FVC ratio, median (p25; p75)	1.0 (0.9; 1.0)	1.0 (0.9; 1.0)	1.0 (0.9; 1.1)	0.590
**DLCO (%), median (p25; p75)** **DLCO <80%, n (%)**	**79.0 (71.5; 93.5)**	**87.0 (75.5; 100.5) 18 (34.6)**	**74.5 (65.0; 81.0) 34 (65.4)**	**0.001 <0.0001**
**6MWT distance, mean (± SD)**	**557.0 (492.6; 610.0)**	**570.0 (523.4; 632.5)**	**516.0 (452.7; 598.6)**	**0.036**
**Pathologic CT, n (%)**	**56 (52.8)**	**24 (42.1)**	**32 (65.3)**	**0.017**

Note: 6MWT, 6-minute walk test; CT, chest-computed tomography; DLCO, diffusion capacity of the lung for carbon monoxide; FEV1, forced expiratory volume in the first second; FVC, forced vital capacity; SD, Standard deviation.

According to multivariate analysis, factors associated with severity were DLCO <80% (OR 5.37; 95% CI 2.05-14.03; p 0.001) and serum CDCP1 (OR 3.85; 95% CI 1.46-10.17; p = 0.006).

Because a relationship between CDCP1 and the profibrotic cytokine TGFb1 has been described [14], we evaluated this relationship in both serum and sputum samples. Regardless of severity, no differences in serum samples were observed (data not shown). However, there were significant differences in induced sputum samples between cases of mild/moderate and severe disease (p <0.0001 for both) (Supplementary Figure 2). No differences in serum CDCP1 values according to severity or days since symptom onset were found (Supplementary Figure 3).

**Figure 1:**
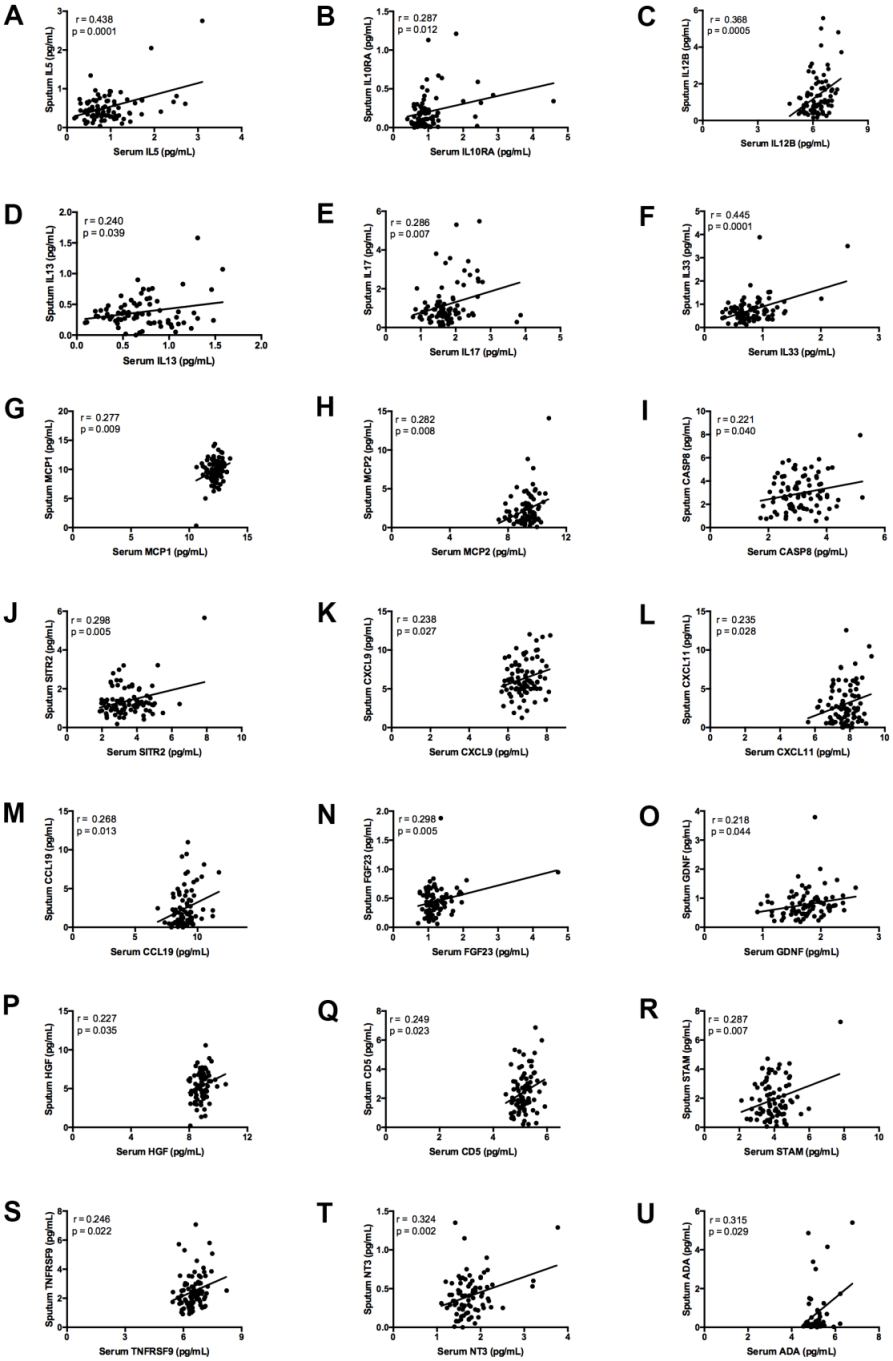
**Graphs showing correlation between plasma and induced sputum levels of statistically significant biomarkers.** Measurement of: (**A**) IL5; (**B**) IL10RA; (**C**) IL12B; (**D**) IL13; (**E**) IL17; (**F**) IL33; (**G**) MCP1; (**H**) MCP2; (**I**) CASP8; (**J**) SIRT2; (**K**) CXCL9; (**L**) CXCL11; (**M**) CCL19; (**N**) FGF23; (**O**) GDNF; (**P**) HGF; (**Q**) CD5; (**R**) STAM; (**S**) TNFRSF9; (**T**) NT3; (**U**) ADA. The continuous line indicates the correlation between the two variables.

Finally, after testing relationships between biomarkers in the serum and induced sputum samples, there were significant associations for 19 proteins (Figure 1). Variables with a p value <0.01 were interleukin (IL) 5 (p = 0.0001), IL33 (p = 0.0001), IL12B (p = 0.0005), neurotrophin 3 (NT3) (p = 0.002), sirtuin2 (SIRT2) (p = 0.005), fibroblast growth Factor 23 (FGF23) (p = 0.005), IL17 (p = 0.007), signal transducing adapter molecule 1 (STAM) (p = 0.007), monocyte chemoattractant protein 2 (MCP2; also known as CCL8) (p = 0.008), and MCP1 (also known as CCL2) (p = 0.009). Direct interactions between cytokines (IL5, IL12B, IL17, IL33) and especially between chemokines (MCP1/CCL2 and MCP2/CCL8) were predicted by STRING analysis (Supplementary Figure 1).

## DISCUSSION

We screened a large panel of biomarkers in serum and induced sputum samples from individuals who had recovered from COVID-19 to investigate post-COVID-19 lung sequelae. Although the SARS [15] and MERS [16] outbreaks affected far fewer people than the current COVID-19 pandemic, it is important to note that up to 33% of patients with MERS [4] and 62% with SARS [17] developed pulmonary fibrosis. Unlike SARS [18] and MERS [19], COVID-19 appears to affect not only the respiratory system but also multiple other systems [20].

Elevated levels of cytokines such as IL1B, IL7, IL8, IL9, and IL10, monocyte chemoattractant protein and tumor necrosis factor (TNF), among others, in COVID-19 have been reported and elevated proinflammatory cytokine levels have been found to correlate with disease severity [21]. Similarly, elevated IL1, IL6, IL8, IL12, TGFb1, CCL2, CXCL9, and CXCL10 are detected in individuals with severe SARS [22]. Although serum proinflammatory cytokines (IL6, IL18) were detected in severe cases, we did not observe differences after comparing patients with mild/moderate and severe cases of COVID-19 in multivariate analysis, suggesting that differences do not persist after recovery. In fact, multivariate analysis only showed differences in serum levels of CDCP1. To our knowledge, no data have been reported about serum CDCP1 and the severity of COVID-19.

CDCP1 (also known as CD318, TRASK, SIMA135, or gp140) is a cell surface glycoprotein expressed in multiple cell types, including lung epithelial cells, hepatocytes, and hematopoietic progenitor cells [14, 23, 24]. CDCP1 is present on interstitial fibroblasts, but not myofibroblasts, in normal lungs and those with idiopathic pulmonary fibrosis [14]. In COVID-19-infected children who developed acute vasculitis, CDCP1 was one of the most significantly upregulated genes [25], but this complication was not observed in our study. The reason for the significant increase in CDCP1 levels in serum, but not in induced sputum samples, is unknown, but increased levels of CDCP1 have been described in patients with autoimmune endocrine diseases [26] and neuroinflammatory states [27], among others. Serum CDCP1 levels in post-COVID patients were lower than those observed in the literature in healthy controls (3.18 NPX) [27].

Microinjuries to the bronchial and/or alveolar epithelium cause the release of growth factors, such as TGFb, which has profibrotic potential, leading to the loss of respiratory capacity [14, 28]. TGFb1 overproduction has been recognized as the most relevant factor related to the progression of pulmonary fibrosis [29]. Shen et al. [30] proposed that the cytokine storm and pathogenesis of COVID-19 are consequence of an unbalanced cytokine network due to increased TGFb activity. These same authors [30] considered that many of the clinical manifestations of COVID-19 (fatigue, dry cough, loss of olfactory and taste, etc.) are related to an increase in TGFb activity. For these reasons, TGFb has been proposed as a therapeutic target for COVID-19 [31]. *In vitro*, TGFb1 decreases CDCP1 expression. CDCP1-depleted cells show upregulation of collagen V and smooth muscle actin (SMA) and further strong enhancement of the effects of TGFb1 on collagen III, collagen V, and SMA [14]. Some authors have reported that CDCP1 is one of the main proteins downregulated by TGFb1 [32], and it has been suggested that CDCP1 is a negative regulator of TGFb1 signaling in fibroblast-to-myofibroblast differentiation via potential CDCP1/TGFb1 crosstalk [14]. Hence, we were surprised to observe a positive relationship between CDCP1 and TGFb1, regardless of severity, after analyzing sputum samples. It will be necessary to carry out more studies and, in the longer term, to determine the real impact of these findings.

By evaluating relationships between pulmonary inflammatory status (in induced sputum samples) and serum protein levels, which in our opinion has not been conducted thus far, we observed that only a few biomarkers (20% of all those evaluated) showed a correlation between serum and induced sputum samples. Sputum induction, a noninvasive method, has been used for studying bronchial inflammation in different respiratory diseases [33]. This technique allows for obtaining small sputum macrophages that exhibit features of highly active inflammatory cells and may therefore be used to analyze inflammatory biomarkers [34]. In relation to SARS-CoV-2 infection, we found a statistical correlation for some cytokines and chemokines. This was especially important for CCL2, which showed main interactions with CCL8, IL12B and IL33. According to Szabo et al. [35], CCL2 released by the lung might contribute to lung tissue damage in severe COVID-19 patients, which is why they suggested that CCR2 antagonists be used to prevent lung damage in these patients. Similarly, Blanco-Melo et al. [36] compared postmortem lung samples from males over 60 years of age who did or did not have COVID-19 and observed that the disease induced robust levels of CCL8 and CCL2, among others. This appears to be consistent with the role of monocytes/macrophages in the immunopathogenesis of the disease [36]. Because we observed similar findings and because some of our biomarkers are proinflammatory cytokines and chemokines, further investigations are necessary to understand their potential implications for recovered patients.

Finally, our previous study and other data showed a reduction in DLCO in those recovering from severe COVID-19 [6, 37–39] suggesting the persistence of impaired lung function.

The limitations of our study include the absence of healthy or uninfected people, though it was not the objective of this study because it was focused on evaluating the severity of COVID-19. Another limitation is the relatively small sample size, which might affect the validity of our results and the lack of a control group. In addition, our measures of inflammatory proteins were cross-sectional, and further studies are necessary to investigate the role of CDCP1. However, this study has important strengths, such as the measurement of 92 biomarkers, the use of a noninvasive procedure to collect sputum samples, and comparison between sputum and serum samples.

In conclusion, although the long-term impact of high serum levels of CDCP1 is still unknown, we should be alert to the potential implications for lung disease. For this reason, it is necessary to follow such patients for longer periods of time to detect and adequately treat potential pulmonary sequelae.

## Supplementary Material

Supplementary Figures

Supplementary Table 1
