# Supplementary material for: Elevated levels of serum CDCP1 in individuals recovering from severe COVID-19 disease
Source: Aging (Albany NY). 2022 Feb 16;14(4):1597–610. doi: 10.18632/aging.203898 (PMC8908919; doi:10.18632/aging.203898)
Supplement: Supplementary Figures [file aging-14-203898-s001.pdf]

## SUPPLEMENTARY FIGURES

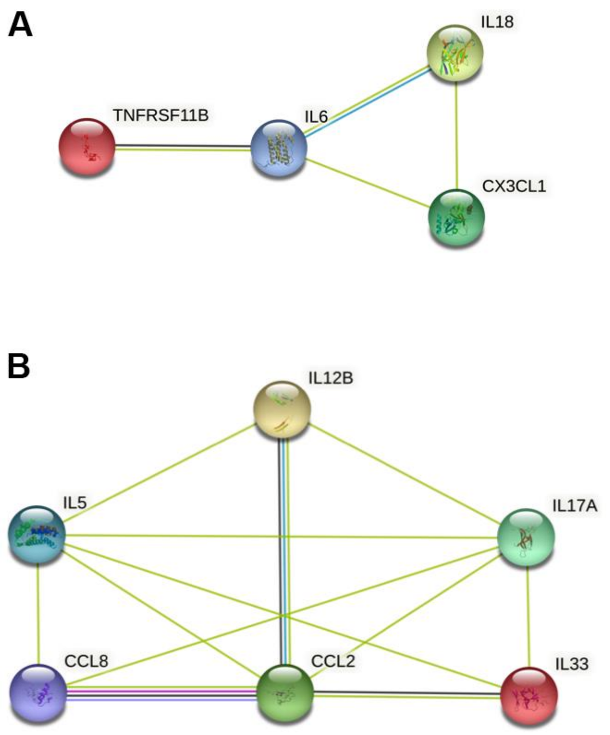


**Supplementary Figure 1. STRING analysis was conducted to map predicted interactions.** (**A**) The resulting network shows several clusters, especially from IL6 with both IL18 and osteoprotegerin/tumor necrosis factor receptor superfamily member 11B (OPG/TNFRSF11B); (**B**) the resulting network shows several clusters, especially between MCP1/CCL2 and MCP2/CCL8.

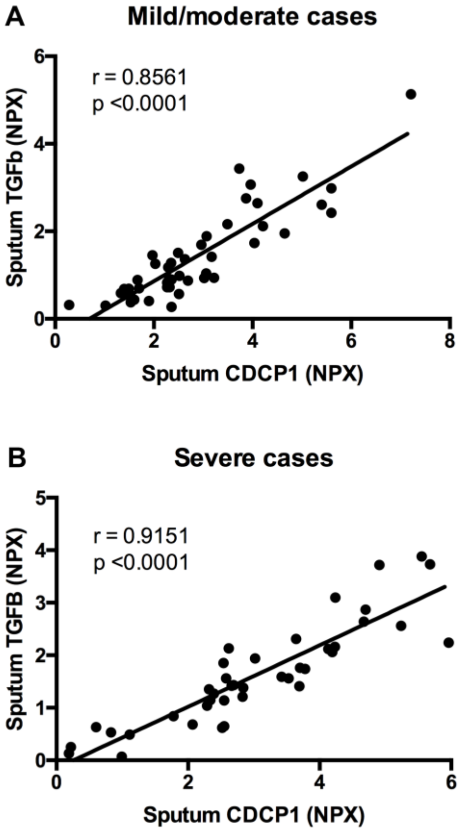


**Supplementary Figure 2.** Graphs showing the correlation between CDCP1 and TGFb1/L levels in sputum samples from patients with mild/moderate (**A**) and severe disease (**B**). The continuous line indicates the correlation between the two variables.

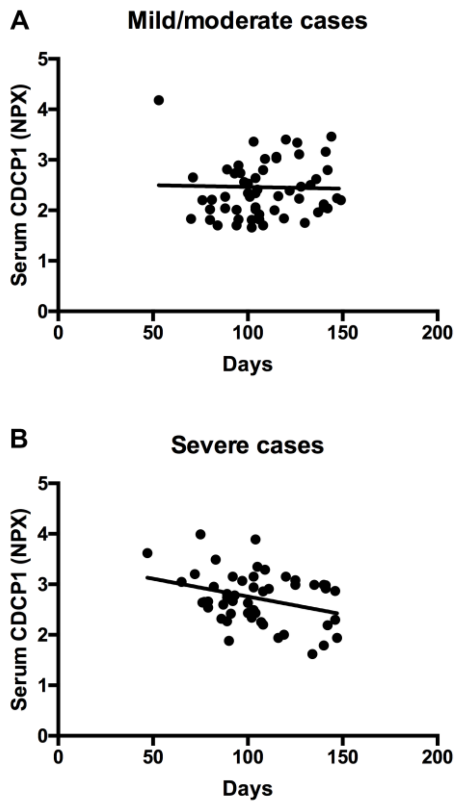


**Supplementary Figure 3.** Graphs showing the correlation between serum CDCP1 and days since symptom onset in patients with mild/moderate (**A**) and severe disease (**B**). The continuous line indicates the correlation between the two variables.
