# Supplementary material for: Elevated levels of serum CDCP1 in individuals recovering from severe COVID-19 disease
Source: Aging (Albany NY). 2022 Feb 16;14(4):1597–610. doi: 10.18632/aging.203898 (PMC8908919; doi:10.18632/aging.203898)
Supplement: Supplementary Table 1 [file aging-14-203898-s002.pdf]

SUPPLEMENTARY TABLE

Supplementary Table 1. Olink® inflammation panel – list of proteins measured in this study.

• Adenosine deaminase (ADA)<br>• Artemin (ARTN)<br>• Axin-1 (AXIN1)<br>• Beta-nerve growth factor (Beta-NGF)<br>• Caspase 8 (CASP-8)<br>• C-C motif chemokine 4 (CCL4)<br>• C-C motif chemokine 19 (CCL19)<br>• C-C motif chemokine 20 (CCL20)<br>• C-C motif chemokine 23 (CCL23)<br>• C-C motif chemokine 25 (CCL25)<br>• C-C motif chemokine 28 (CCL28)<br>• CD40L receptor (CD40)<br>• CUB domain-containing protein 1 (CDCP1)<br>• C-X-C motif chemokine 1 (CXCL1)<br>• C-X-C motif chemokine 5 (CXCL5)<br>• C-X-C motif chemokine 6 (CXCL6)<br>• C-X-C motif chemokine 9 (CXCL9)<br>• C-X-C motif chemokine 10 (CXCL10)<br>• C-X-C motif chemokine 11 (CXCL11)<br>• Cystatin D (CST5)<br>• Delta and Notch-like epidermal growth factor-related receptor (DNER)<br>• Eotaxin-1 (CCL11)<br>• Eukaryotic translation initiation factor 4E-binding protein 1 (4E-BP1)<br>• Fibroblast growth factor 5 (FGF-5)<br>• Fibroblast growth factor 19 (FGF-19)<br>• Fibroblast growth factor 21 (FGF-21)<br>• Fibroblast growth factor 23 (FGF-23)<br>• Fms-related tyrosine kinase 3 ligand (Flt3L)<br>• Fractalkine (CX3CL1)<br>• Glial cell line-derived neurotrophic factor (GDNF)<br>• Hepatocyte growth factor (HGF)<br>• Interferon gamma (IFN-gamma)<br>• Interleukin-1 alpha (IL-1 alpha)<br>• Interleukin-2 (IL-2)<br>• Interleukin-2 receptor subunit beta (IL-2RB)<br>• Interleukin-4 (IL-4)<br>• Interleukin-5 (IL-5)<br>• Interleukin-6 (IL-6)<br>• Interleukin-7 (IL-7)<br>• Interleukin-8 (IL-8)<br>• Interleukin-10 (IL-10)<br>• Interleukin-10 receptor subunit alpha (IL-10RA)<br>• Interleukin-10 receptor subunit beta (IL-10RB)<br>• Interleukin-12 subunit beta (IL-12B)<br>• Interleukin-13 (IL-13)<br>• Interleukin-15 receptor subunit alpha (IL-15RA)<br>• Interleukin-17A (IL-17A)<br>• Interleukin-17C (IL-17C)<br>• Interleukin-18 (IL-18)	• Interleukin-18 receptor 1 (IL-18R1)<br>• Interleukin-20 (IL-20)<br>• Interleukin-20 receptor subunit alpha (IL-20RA)<br>• Interleukin-22 receptor subunit alpha-1 (IL-22 RA1)<br>• Interleukin-24 (IL-24)<br>• Interleukin-33 (IL-33)<br>• Latency-associated peptide transforming growth factor beta 1 (LAP TGF-beta-1)<br>• Leukemia inhibitory factor (LIF)<br>• Leukemia inhibitory factor receptor (LIF-R)<br>• Macrophage colony-stimulating factor 1 (CSF-1)<br>• Macrophage inflammatory protein 1-alpha (CCL3)<br>• Matrix metalloproteinase-1 (MMP-1)<br>• Matrix metalloproteinase-10 (MMP-10)<br>• Monocyte chemotactic protein 1 (MCP-1)<br>• Monocyte chemotactic protein 2 (MCP-2)<br>• Monocyte chemotactic protein 3 (MCP-3)<br>• Monocyte chemotactic protein 4 (MCP-4)<br>• Natural killer cell receptor 2B4 (CD244)<br>• Neurotrophin-3 (NT-3)<br>• Neurturin (NRTN)<br>• Oncostatin-M (OSM)<br>• Osteoprotegerin (OPG)<br>• Programmed cell death 1 ligand 1 (PD-L1)<br>• Protein S100-A12 (EN-RAGE)<br>• Signaling lymphocytic activation molecule (SLAMF1)<br>• SIR2-like protein 2 (SIRT2)<br>• STAM-binding protein (STAMPB)<br>• Stem cell factor (SCF)<br>• Sulfotransferase 1A1 (ST1A1)<br>• T-cell surface glycoprotein CD5 (CD5)<br>• T-cell surface glycoprotein CD6 isoform (CD6)<br>• T-cell surface glycoprotein CD8 alpha chain (CD8A)<br>• Thymic stromal lymphopoietin (TSLP)<br>• TNF-beta (TNFB)<br>• TNF-related activation-induced cytokine (TRANCE)<br>• TNF-related apoptosis-inducing ligand (TRAIL)<br>• Transforming growth factor alpha (TGF-alpha)<br>• Tumor necrosis factor (Ligand) superfamily, member 12 (TWEAK)<br>• Tumor necrosis factor (TNF)<br>• Tumor necrosis factor ligand superfamily member 14 (TNFSF14)<br>• Tumor necrosis factor receptor superfamily member 9 (TNFRSF9)<br>• Urokinase-type plasminogen activator (uPA)<br>• Vascular endothelial growth factor A (VEGF-A)
